# Supplementary material for: In vivo transomic analyses of glucose-responsive metabolism in skeletal muscle reveal core differences between the healthy and obese states
Source: Sci Rep. 2022 Aug 12;12:13719. doi: 10.1038/s41598-022-17964-9 (PMC9374747; doi:10.1038/s41598-022-17964-9)
Supplement: Supplementary file 1 — Supplementary Information 1. [file 41598_2022_17964_MOESM1_ESM.pdf]

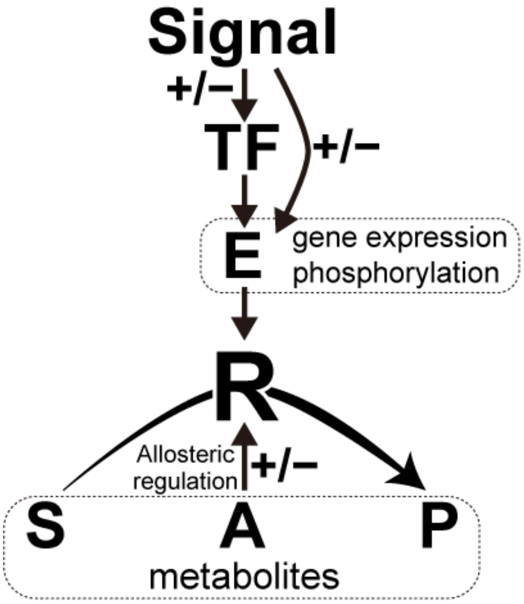

Fig. S1. The regulatory network for metabolic reactions. A generic metabolic reaction (R) is catalyzed by a metabolic enzyme (E) and involves metabolites that function as the substrate (S), product (P), or allosteric regulator (A). For reversible reactions, the product is also a substrate and the substrate is also a product (not shown). Positive and negative signs indicate positive and negative regulation, respectively. Regulation of a metabolic reaction by a metabolic enzyme consists of regulation by changing the amount of enzyme through gene expression and regulation by changing enzyme activity through posttranslational modifications, in particular phosphorylation. Gene expression is regulated by one or more transcription factors (TFs) and signaling molecules (Signals) regulate both transcription factor activity and metabolic enzyme

activity by changing the phosphorylation status. This figure was modified from Supplementary Figure 1 of Kokaji et al. (2020).

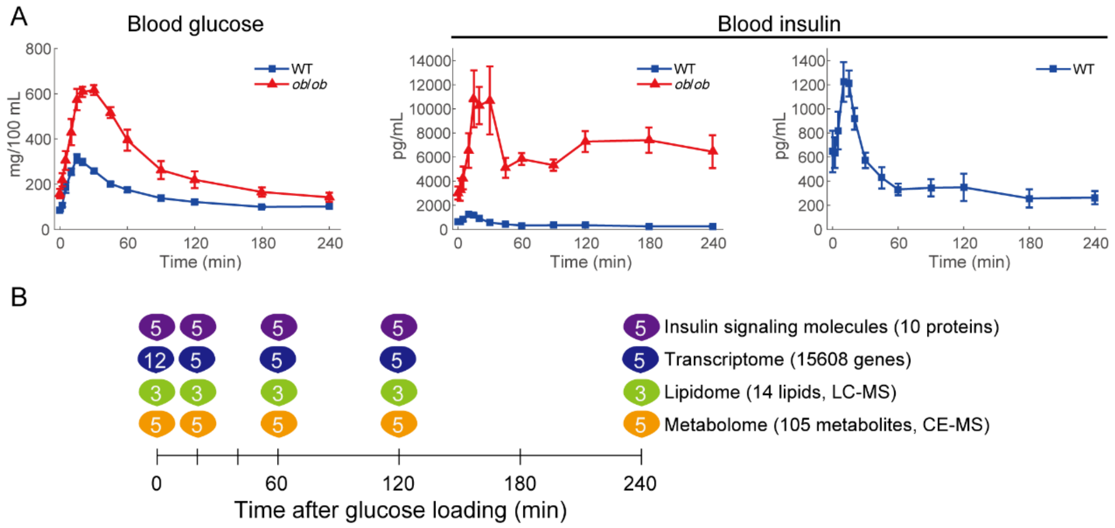

Fig. S2. Oral glucose administration and multiomic measurements. (**A**) Blood glucose and blood insulin of WT mice (blue) and *ob*/*ob* mice (red) during oral glucose administration. The data of blood glucose and insulin levels measured in our previous study are shown (Kokaji et al., 2020). The means and SEMs of five mice per genotype are shown. (**B**) We orally administered glucose to 16 h-fasting WT and *ob*/*ob* mice, and collected the skeletal muscle at 0, 20, 60, 120, and 240 min after administration. We performed metabolomics, transcriptomics, and Western blotting for the phosphorylation of insulin signaling molecules in the skeletal muscle. The number of mice per genotype in each measurement is shown at each time point. This figure was modified from Supplementary Figure 2 of Kokaji et al. (2020).

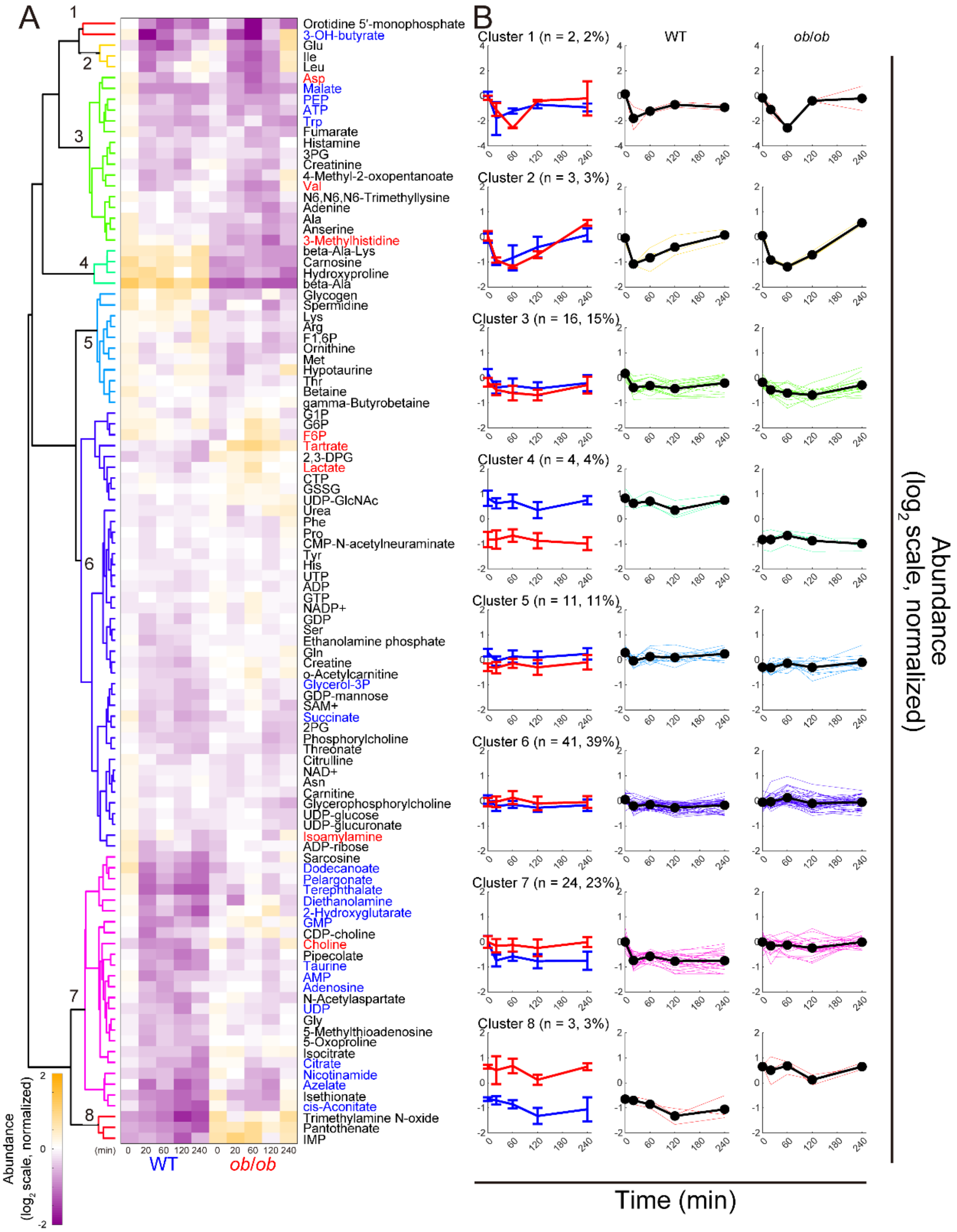

Fig. S3. Hierarchical clustering of time courses of metabolites in the skeletal muscle. (**A**) The heat map and hierarchical clustering of the time courses of metabolites in the skeletal muscles of WT and *ob*/*ob* mice following oral glucose administration.

The colors of and numbers on tree diagram indicate the cluster of each metabolite. To investigate the changes from fasting state, two time courses of each metabolite were divided by the geometric mean of the values of WT mice and *ob*/*ob* mice in fasting state (0 min), and then $\log_2$-transformed. The colors of the names of metabolites indicate WT mice-specific glucose-responsive metabolites (blue), *ob*/*ob* mice-specific glucose-responsive metabolites (red), and metabolites that are not glucose-responsive (black). (**B**) Averaged time courses of the metabolites for all eight clusters. Left panel shows averaged time courses of the metabolites as the mean and standard deviation in a cluster for WT mice (blue) and *ob*/*ob* mice (red). Middle panel (WT mice) and right panel (*ob*/*ob* mice) show average (thick line) and individual (thin line) time courses of the metabolites in a cluster in WT or *ob/ob* mice.

Clusters 1, 2, and 3 was comprised of metabolites which were decreased in both WT and *ob/ob* mice, and the responses in cluster 1 were largest of the three clusters. Orotidine 5'-monophosphate and 3-OH-butyrate was classified in this cluster. The responses in cluster 2 were larger than those in cluster 3. Cluster 2 consisted of three amino acids; valine, leucine, and glutamate. Other amino acids (aspartate, valine, alanine, and tryptophan), downstream metabolites of the glycolytic pathway (3-phosphoglyceric acid [3PG] and phosphoenolpyruvate [PEP]) and metabolites of the

TCA cycle (fumarate and malate) were classified into cluster 3. Cluster 4 included metabolites that were more abundant in WT mice at all timepoints. This cluster mainly comprised β-alanine, carnosine, a dipeptide of β-alanine and histidine, and β-alanine-lysine. Metabolites in cluster 5 were also more abundant in WT mice; however, the difference was smaller compared to cluster 4. This cluster mainly comprised amino acids such as lysine, arginine, threonine, and ornithine. Fructose 1,6-bisphosphate (F1,6BP) and glycogen was also classified into cluster 5. Metabolites in cluster 6 were observed at slightly higher levels in *ob/ob* mice, and showed almost no changes by glucose administration. Many amino acids, metabolites of the central carbon metabolism, and nucleic acids were classified into this cluster. Metabolites of the glycolytic pathway, such as glucose-1-phosphate (G1P), glucose-6-phosphate (G6P), fructose 6-phosphate (F6P), and lactate were also included. Among these, F6P and lactate were significantly increased only in *ob/ob* mice. Metabolites in cluster 7 tended to decrease specifically in WT mice (14/24 metabolites showed significant decreases). Metabolites of the TCA cycle, such as citrate, isocitrate, and cis-aconitate, and nucleic acids (adenosine monophosphate [AMP], guanosine monophosphate [GMP], and adenosine) were included in this cluster. Metabolites in cluster 8 (inosine

monophosphate, pantothenate, and trimethylamine N-oxide) were abundant in *ob/ob* mice compared to WT mice, and the difference was quite large.

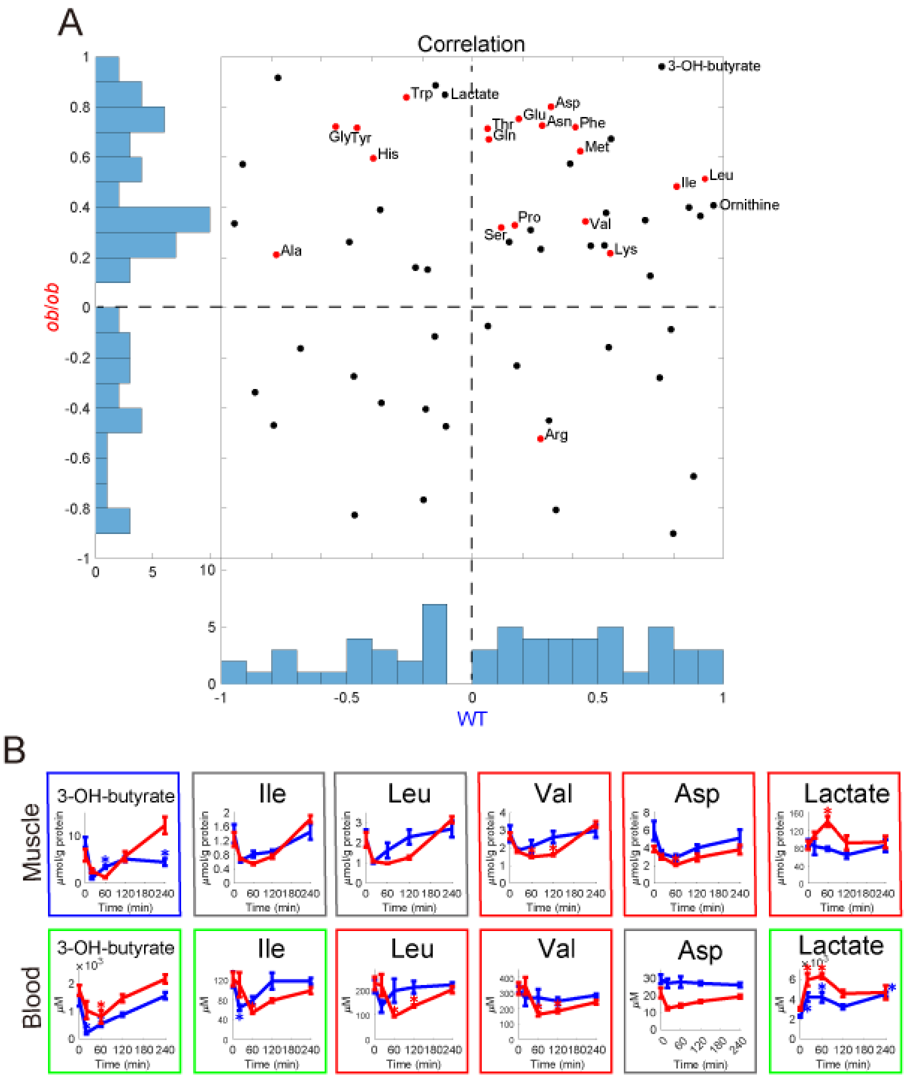

Fig. S4. Time courses of metabolite changes in the skeletal muscle and in the blood.

(**A**) Histograms and scatter plot of Pearson's correlation coefficients between the time courses of changes in metabolites measured in skeletal muscle and blood in WT mice and *ob*/*ob* mice. Red dots indicate 19 proteogenic amino acids measured in both skeletal muscle and blood. (**B**) Time courses of changes in the indicated metabolites in the skeletal muscle and blood of WT mice (blue) and *ob*/*ob* mice (red) following oral

glucose administration. The means and SEMs of five mice per genotype are shown. The colors of the frames indicate common glucose-responsive metabolites (green), WT-specific glucose-responsive metabolites (blue), *ob*/*ob*-specific glucose-responsive metabolites (red), and not glucose-responsive metabolites either in WT mice or in *ob*/*ob* mice (gray). *q value < 0.1 and absolute $\log_2$ fold change > 0.585.

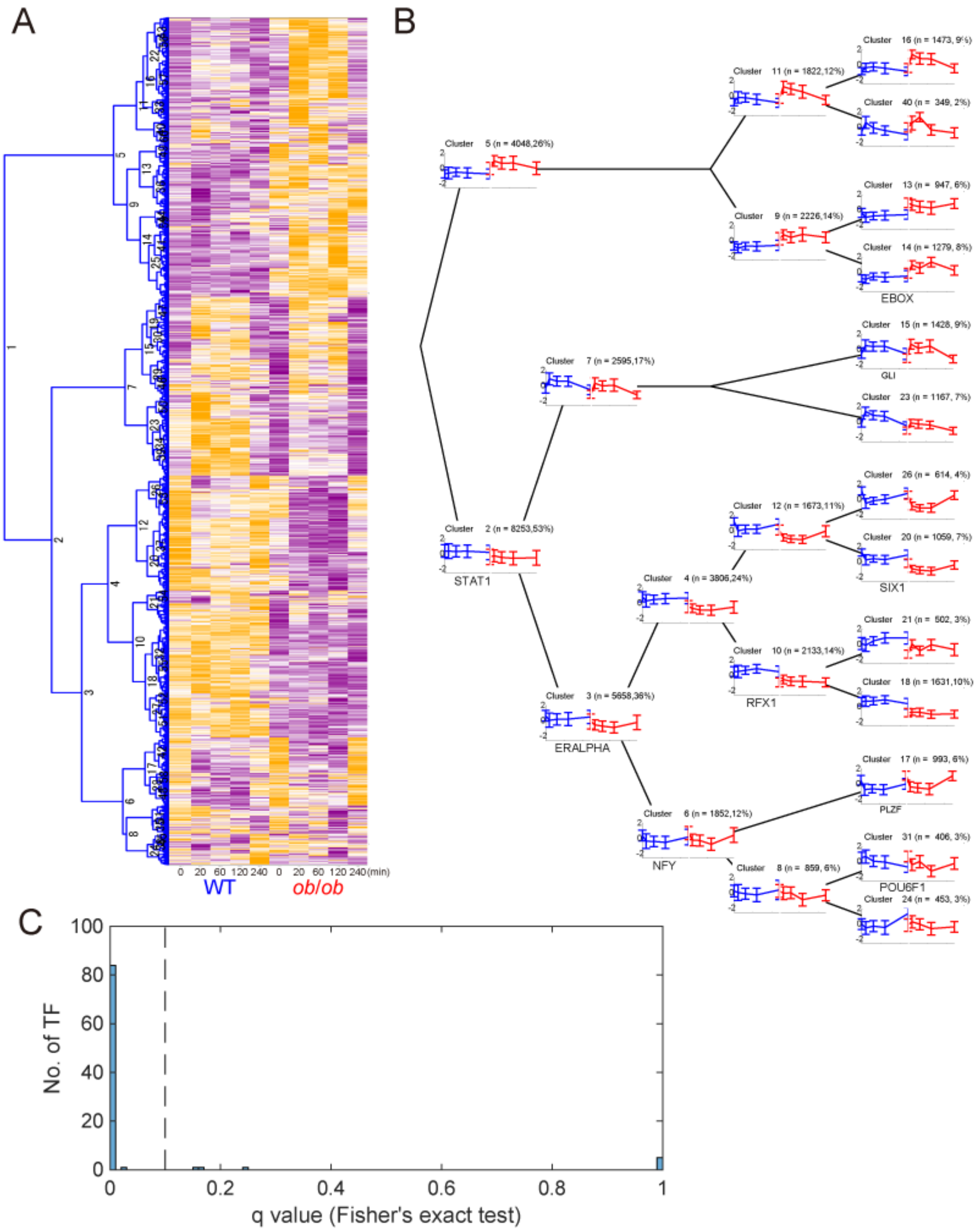

Fig. S5. Hierarchical clustering of the time courses of gene expression in the skeletal muscle and inference of regulatory connections between transcription factors and genes. (**A**) The heat map and hierarchical clustering of the Z-score

normalized time courses of gene expressions in the skeletal muscle of WT and *ob*/*ob* mice following oral glucose administration. The hierarchical clustering was performed using Euclidean distance and Ward's method. The numbers on the tree diagram indicates the cluster identity. Each cluster includes only the genes that show a significant response at any time point either in WT mice or *ob*/*ob* mice or significant differences between WT mice and *ob*/*ob* mice before glucose administration (0 min). (**B**) The averaged time courses of the gene expression for each cluster of WT mice (blue) and *ob*/*ob* mice (red). The mean and standard deviation of the time courses of gene expressions in the cluster are shown. The time courses are presented on the tree diagram of hierarchical clustering. Significantly enriched transcription factor motifs (q value < 0.1) in the cluster are described with the time courses. According to the enriched transcription factor motifs, we defined the regulatory connections between the transcription factors and the genes in the cluster. To avoid overestimation, we excluded a cluster from the inference if the transcription factor binding motif was more enriched in the children clusters. The remaining transcription factor motifs, but not the excluded transcription factor motifs, are described here. The transcription factor motifs enriched in the upstream clusters are not described in the downstream clusters. (**C**) The histogram of the q values for the overlaps between the inferred genes of the

transcription factors and those predicted from ChIP data. The ChIP data were obtained from the ChIP-Atlas database (Oki et al., 2018). The q values were calculated by the one-tailed Fisher's exact test and Benjamini–Hochberg procedure (Yoav Benjamini, 1995).

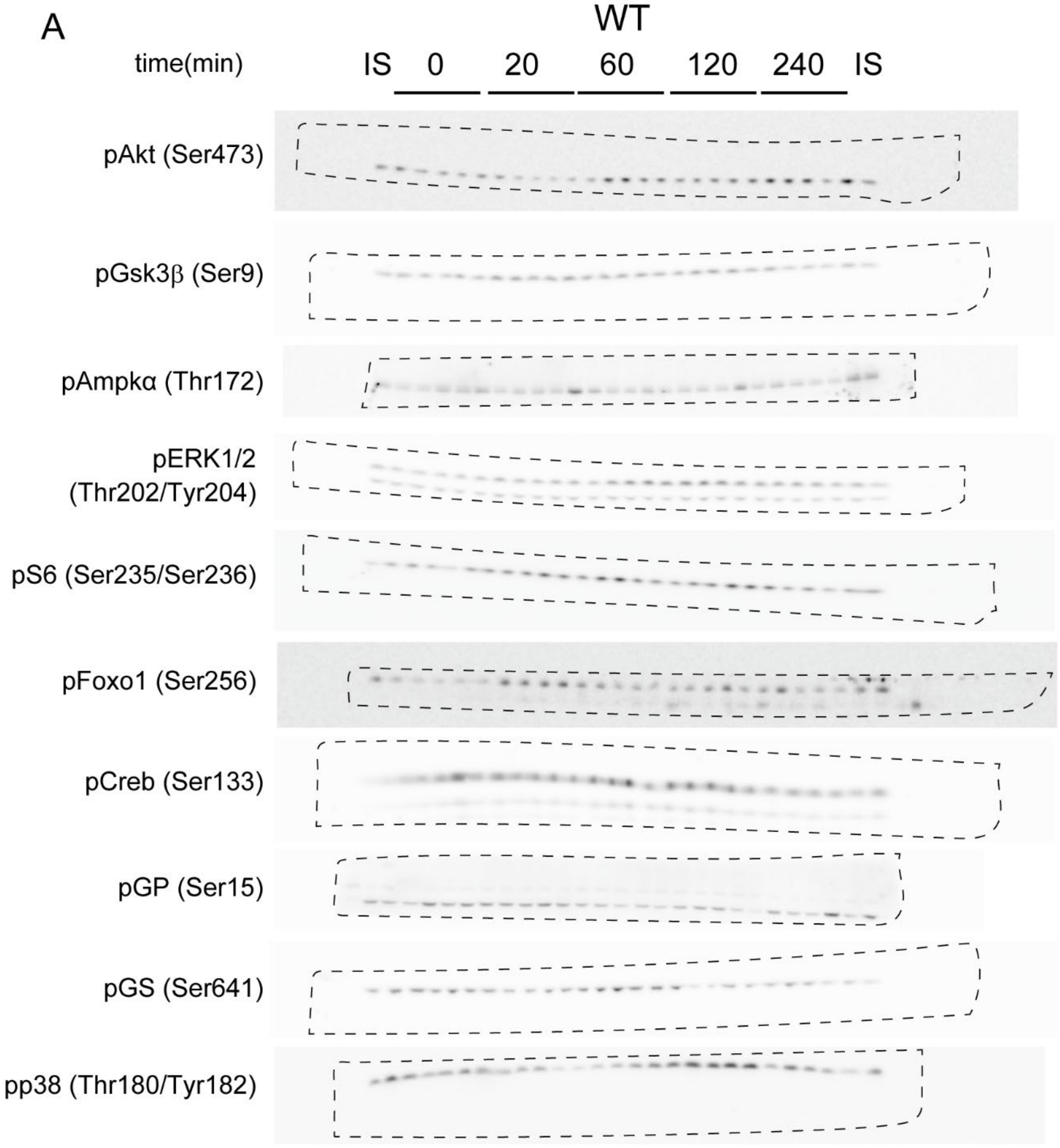
A
WT
time(min)
IS
0
20
60
120
240
IS
pAkt (Ser473)
pGsk3β (Ser9)
pAmpkα (Thr172)
pERK1/2
(Thr202/Tyr204)
pS6 (Ser235/Ser236)
pFoxo1 (Ser256)
pCreb (Ser133)
pGP (Ser15)
pGS (Ser641)
pp38 (Thr180/Tyr182)

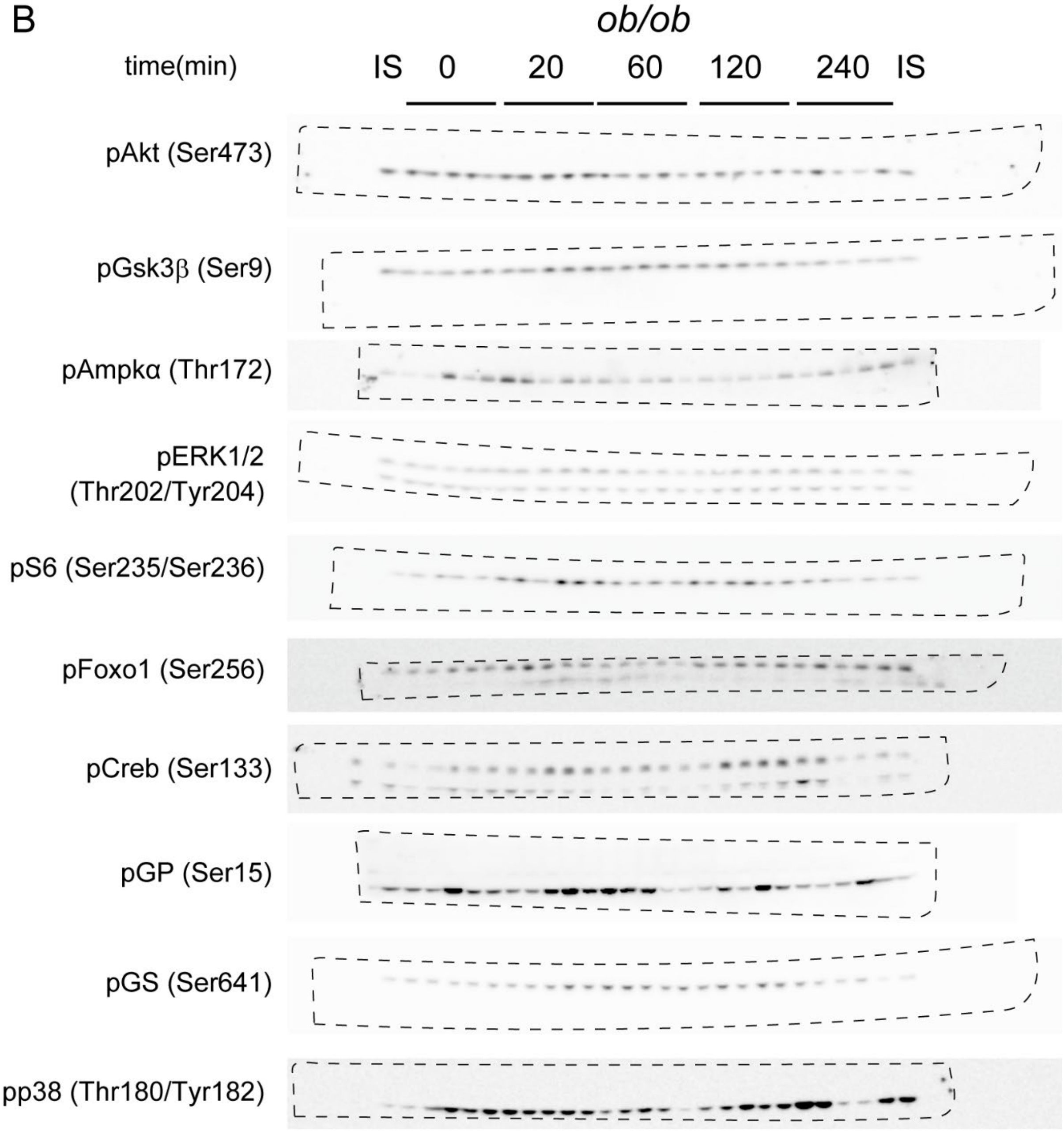

Fig. S6. Western blotting for insulin signaling molecules. The phosphorylation of the indicated insulin signaling molecules in the skeletal muscle of WT (A) and *ob*/*ob* (B) mice at the indicated time point after oral glucose administration. Residues in parentheses indicate the phosphorylation site(s) (human sequence numbering)

recognized by the antibodies. The membranes were cut prior to hybridisation with antibodies. Dashed lines indicate the edges of the cut membrans. Western blot data for all mice are shown (n = 5 mice per genotype for glucose administration).

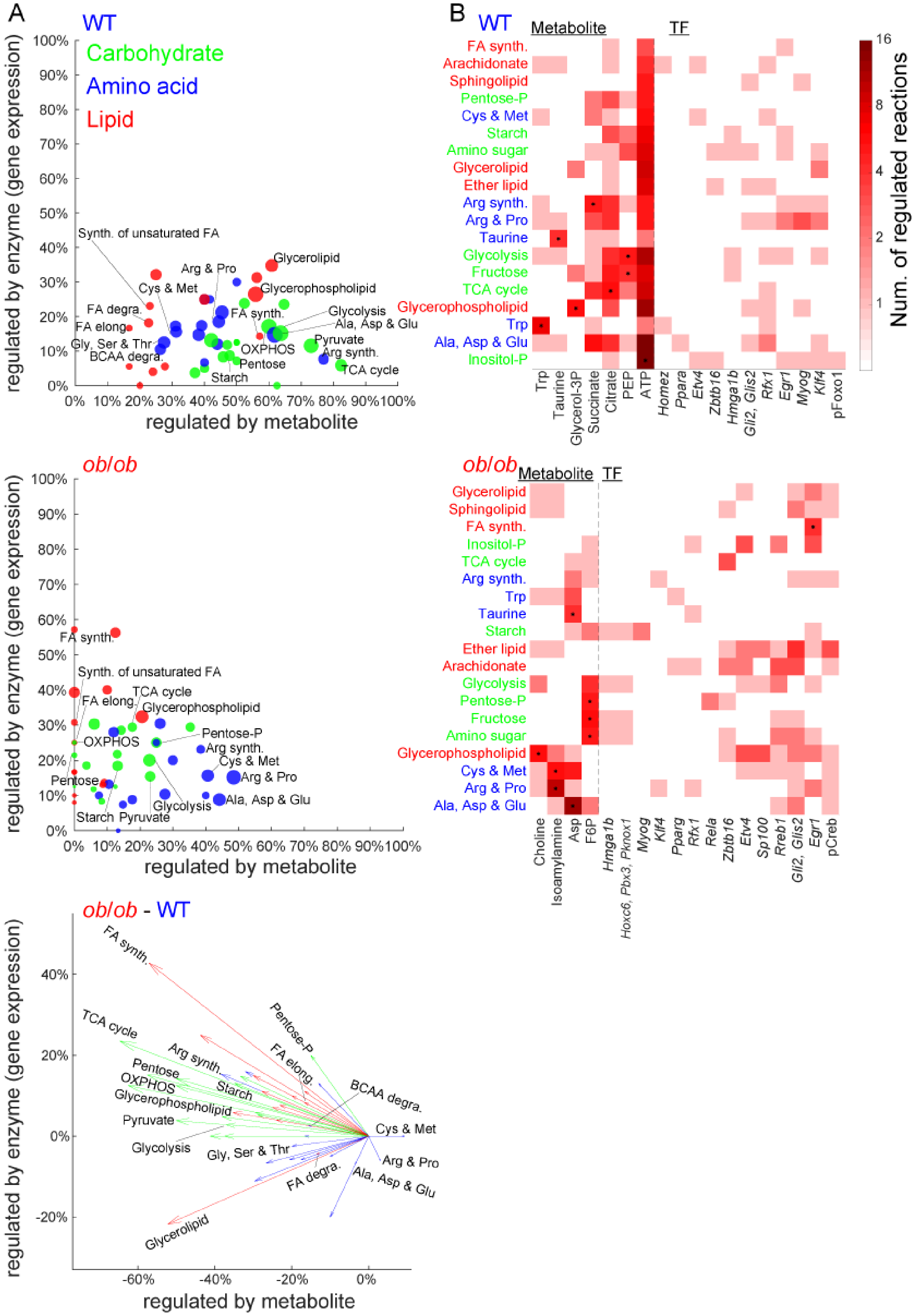
A
WT
Carbohydrate
Amino acid
Lipid
regulated by enzyme (gene expression)
regulated by metabolite
Synth. of unsaturated FA
Glycerolipid
Arg & Pro
Cys & Met
Glycerophospholipid
FA degra.
FA elong.
FA synth.
Glycolysis
Ala, Asp & Glu
Gly, Ser & Thr
BCAA degra.
OXPHOS
Pyruvate
Arg synth.
Pentose
Starch
TCA cycle
B
WT
Metabolite
TF
FA synth.
Arachidonate
Sphingolipid
Pentose-P
Cys & Met
Starch
Amino sugar
Glycerolipid
Ether lipid
Arg synth.
Arg & Pro
Taurine
Glycolysis
Fructose
TCA cycle
Glycerophospholipid
Trp
Ala, Asp & Glu
Inositol-P
Trp
Taurine
Glycerol-3P
Succinate
Citrate
PEP
ATP
Homez
Ppara
Etv4
Zbtb16
Hmga1b
Gli2, Glis2
Rfx1
Egr1
Myog
Klf4
pFoxo1
Num. of regulated reactions
ob/ob
FA synth.
Synth. of unsaturated FA
TCA cycle
FA elong.
Glycerophospholipid
Pentose-P
OXPHOS
Arg synth.
Cys & Met
Arg & Pro
Pentose
Ala, Asp & Glu
Glycolysis
Starch
Pyruvate
ob/ob
Metabolite
TF
Glycerolipid
Sphingolipid
FA synth.
Inositol-P
TCA cycle
Arg synth.
Trp
Taurine
Starch
Ether lipid
Arachidonate
Glycolysis
Pentose-P
Fructose
Amino sugar
Glycerophospholipid
Cys & Met
Arg & Pro
Ala, Asp & Glu
Choline
Isoamylamine
Asp
F6P
Hmga1b
Hoxc6, Pbx3, Pknox1
Myog
Klf4
Pparg
Rfx1
Rela
Zbtb16
Etv4
Sp100
Rreb1
Gli2, Glis2
Egr1
pCreb
ob/ob - WT
FA synth.
Pentose-P
TCA cycle
FA elong.
Arg synth.
BCAA degra.
Pentose
Starch
OXPHOS
Glycerophospholipid
Cys & Met
Pyruvate
Glycolysis
Gly, Ser & Thr
Arg & Pro
FA degra.
Ala, Asp & Glu
Glycerolipid

Fig. S7. Metabolic reactions regulated by glucose-responsive molecules in each metabolic pathway node. (**A**) For each metabolic pathway node, the percentage of regulated metabolic reactions by glucose-responsive metabolites (*x*-axis) and glucose-responsive genes encoding metabolic enzymes (*y*-axis) was plotted for WT and *ob/ob* mice. The size of the dots indicates the number of regulated metabolic reactions in each metabolic pathway node either by glucose-responsive metabolites, genes, or both. The colors of the dots indicate the classes of metabolic pathway node according to the KEGG database [18,19]: carbohydrate (green), amino acid (blue), and lipid (red). (**B**) Heat maps showing the number of regulated metabolic reactions in each metabolic pathway node (rows) by each glucose-responsive metabolite (left columns) and each transcription factor-dependent glucose-responsive genes of metabolic enzymes (right columns) in WT and *ob/ob* mice. The * symbols indicate significant associations (q value < 0.01) between metabolic reactions in the metabolic pathway node and those regulated by glucose-responsive molecules (Data File S10). The q values were calculated by the one-tailed Fisher's exact test and Benjamini–Hochberg procedure (Yoav Benjamini, 1995). Only metabolic pathway nodes with significant associations with any glucose-responsive molecule are shown. Only glucose-responsive metabolites with significant associations with any metabolic pathway node are shown.

Table S1. Pathway enrichment analysis of the glucose-responsive genes.

Parameter	WT mice	*ob*/*ob* mice
Body mass, g	22.5 ± 1.1	42.9 ± 3.0
Blood glucose, mg/dL	84.2 ± 8.58	159 ± 46.2
Blood triglyceraide, mg/dL	88 ± 22.1	54 ± 29.8
Total blood cholesterol, mg/dL	100 ± 10.2	196 ± 48.8
Blood insulin, ng/mL	0.647 ± 0.385	3.02 ± 1.21
Muscle glycogen	9.18 ± 4.23	4.43 ± 1.14

Values are presented as group means ± standard deviation. The data of body mass, blood triglyceride and total blood cholesterol were downloaded from Japan SLC Inc. (Shizuoka, Japan). n = 5 for blood glucose, blood insulin, and muscle glycogen, n = 10 for body mass, blood triglyceride and total blood cholesterol.

Data File S1. Metabolomic data.

Data File S2. Lipidomic data.

Data File S3. Transcriptomic data.

Data File S4. Pathway enrichment analysis of glucose-responsive genes.

Data File S5. Enrichment analysis of gene clusters.

Data File S6. Inferred regulatory connections between transcription factors and genes.

Data File S7. Overlap between the inferred genes of transcription factors and those predicted from experimental ChIP data.

Data File S8. Western blotting data.

Data File S9. Regulatory transomic network for glucose-responsive metabolic reactions.

Data File S10. Significant associations between glucose-responsive molecules and metabolic pathways.
